# Antioxidant Activity of Extracts of Balloon Flower Root (*Platycodon grandiflorum*), Japanese Apricot (*Prunus mume*), and Grape (*Vitis vinifera*) and Their Effects on Beef Jerky Quality

**DOI:** 10.3390/foods13152388

**Published:** 2024-07-28

**Authors:** Beom Joon Kim, Dong Gyun Yim, Martin J. T. Reaney, Young Jun Kim, Youn Young Shim, Suk Nam Kang

**Affiliations:** 1 Department of Animal Resource, Daegu University, Daegu 38453, Republic of Korea; bj-kim@daegu.ac.kr; 2 Department of Animal Science, Kyungpook National University, Sangju 37224, Republic of Korea; tousa0994@naver.com; 3 Department of Animal Science and Biotechnology, Kyungpook National University, Sangju 37224, Republic of Korea; 4 Department of Food and Bioproduct Sciences, University of Saskatchewan, Saskatoon, SK S7N 5A8, Canada; martin.reaney@usask.ca; 5 Prairie Tide Diversified Inc., Saskatoon, SK S7J 0R1, Canada; 6 Department of Food and Biotechnology, Korea University, Sejong 30019, Republic of Korea; yk46@korea.ac.kr

**Keywords:** total polyphenol content, potassium sorbate, vitamin E, natural preservative, thiobarbituric acid reactive substances (TBARSs), sensory analysis

## Abstract

This research examines the total polyphenol and flavonoid content and antioxidant activity of natural ingredients such as balloon flower root extract (BFE), Japanese apricot extract (JAE) and grape extract (GE). In addition, their effect on beef jerky quality characteristics was investigated when the extracts were used as alternatives to potassium sorbate (PS) and vitamin E (VE). BFE had higher (*p* < 0.05) total flavonoid content (TFC) (6.85 mg CAT eq/g), total polyphenol content (TPC) (10.52 mg RUT eq/g), 1,1-diphenyl-2-picrylhydrazyl (DPPH) radical (62.96%), and 2,2′-azino-bis (3-ethylbenzthiazoline-6-sulphonic acid) (ABTS) radical scavenging activity (87.60%) compared to other extracts. Although all extracts showed lower activity than BHT in all antioxidant activity tests, the BFE and JAE showed higher (*p* < 0.05) activity than the GE in the DPPH and FRAP assays. In contrast, in the ABTS assay, both BFE and GE showed increased activity (*p* < 0.05) compared to JAE. The jerky was prepared by adding 0.05% (*v*/*v*) each of BFE, JAE and GE. Furthermore, a control sample of jerky was also prepared by adding 0.10% (*w*/*v*) PS and 0.05% VE, respectively. On day 30, the redness (a*) values of the BFE and PS samples were also found to be significantly higher than those of the other samples (*p* < 0.05). Additionally, the yellowness (b*) values of the BFE sample were also found to be significantly higher than those of the other samples (*p* < 0.05). The thiobarbituric acid reactive substances (TBARSs) on day 30 were lower in the jerky treated with PS, VE, and GE compared to those treated with BFE and JAE (*p* < 0.05). In the sensory analysis, beef jerky with BFE had significantly higher overall acceptability scores on days 1 and 30 (*p* < 0.05). The addition of BFE to beef jerky influenced the increase in a* and b* values on day 30. The addition of GE effectively suppressed lipid oxidation to a level comparable to that of the PS and VE at day 30. Furthermore, the addition of BFE enhanced the overall acceptability of sensory characteristics.

## 1. Introduction

The distribution market for beef is gradually expanding, and with increasing income and improving dietary standards, beef consumption is steadily rising [1]. Consequently, there is growing interest in high-quality meat, and the sale of safe and high-quality products has become crucial [2]. However, forequarters, rounds, and briskets are perceived as less preferred cuts, resulting in stagnant consumption, indicating the need to develop various products to increase their utilization [3,4].

Beef jerky is a processed meat product typically made by slicing beef thinly and then drying it. It is produced in stick form for convenient consumption and sold in smaller, more affordable portions. With rising national income, consumers who once prioritized price and taste now carefully select foods considering the ingredients and nutritional content for health reasons. This shift in consumer perception from quantity to quality has led the food industry to develop numerous products with added extracts. Companies producing beef jerky are also adapting to this change by incorporating natural ingredients, such as lotus leaf extract [5], citron and plum extracts [6], and grapefruit, mandarin, and cinnamon [7] to enhance quality.

In beef jerky, potassium sorbate (PS) is commonly used as a preservative and sodium erythorbate as an antioxidant [5]. Reactive oxygen species (ROS), known to be the direct cause of oxidation, are inevitably generated in respiring organisms [8,9,10]. These highly reactive and unstable free radicals attack macromolecules in the body, causing cancer, cytotoxicity, and mutations, posing problems to both human health and food safety [11,12]. To prevent this, nitrite, which effectively inhibits oxidation and anaerobic bacteria, is added [13], along with antioxidants such as butylated hydroxyanisole (BHA), butylated hydroxytoluene (BHT), and propyl gallate to extend shelf life, and sorbic acid as a preservative [14]. However, nitrites can react with secondary and tertiary amines to form carcinogenic nitroso-amines [15], and high doses of BHA and BHT have been reported to cause hepatomegaly and carcinogenicity [16].

There is ongoing research into natural antioxidants, which are safe for humans and have excellent antioxidant properties, to replace synthetic antioxidants. Most plants contain compounds such as phenolic compounds, vitamin C and vitamin E, which have been shown to have antioxidant activity [17]. The antioxidant substances identified in extracts of Finnish plants also had antimicrobial activity [18]. Most antioxidant substances are isolated for their functional properties, with significant research focused on developing natural materials for pharmaceuticals. Nevertheless, further research is required on natural preservatives and product development. For example, studies have demonstrated the preservative effects of vitamin E as the principal lipid-soluble antioxidant in meat products [19], the use of cactus stem extract in cosmetics [20], and the inhibition of tofu spoilage with smartweed extract [21]. However, to date, very limited information is available on the use of additives, such as balloon flower root, plum, and grape, in jerky.

Therefore, this study aims to investigate the potential application of natural additives in beef jerky by adding balloon flower root, plum, and grape for antioxidant activity and lipid peroxidation inhibition. In addition, the quality characteristics and storage stability of the jerky were evaluated to determine the potential for quality improvement and extended shelf life with natural additives.

## 2. Materials and Methods

### 2.1. Balloon Flower Root Extract (BFE), Japanese Apricot Extract (JAE), and Grape Extract (GE)

For the preparation of the natural extracts in this study, the methodology of Seleshe et al.’s [22] study was modified. The dried samples (50 °C for 1 week) were crushed using a mortar and then passed through a 1 mm sieve size to obtain a fine powder for extraction. The dried powder (5 g) was combined with distilled water (1 L) in a round-bottom flask and extracted at 95 °C for 4 h using a cooling condenser. After extraction, the solutions were filtered through Whatman filter paper No. 1 (GE Healthcare Ltd., New Jersey, NY, USA). The extracts were concentrated using a rotary evaporator (Eyela N-1300V-WB, Tokyo Rikakikai Co. Ltd., Tokyo, Japan) at 45 °C, followed by freeze-drying (Ilsinbiobase, Dongducheon, Republic of Korea), and stored at −20 °C prior to use. The extracts were dissolved in dimethyl sulfoxide (DMSO; Sigma-Aldrich Co., St. Louis, MO, USA) for analysis.

### 2.2. Preparation of Beef Jerky

The experiment utilized the eye of round steak derived from cattle. The frozen round steak was thawed at 4 °C, and blood and connective tissue were removed before use. The jerky was prepared by dividing the curing solution into one with conventional preservatives and one with added extracts according to a modified method based on Lim et al. [23]. The formulation is shown in Table 1. Extracts were added at a level of 0.05% (*v*/*v*), and for extracts with added sugar, the sugar amount was adjusted accordingly. The control group used sodium sorbate and vitamin E (VE), added at 0.10% and 0.05% (*w*/*v*) of the weight, respectively. The beef was sliced to a thickness of 6.5 mm using a meat slicer (Hankook Fujee Industries Co., Ltd., Hwaseong, Republic of Korea) and cured in the prepared solution in a refrigerator at 4 °C for 12 h, turning every 6 h. After curing, the jerky was dried using an automatic smokehouse (SMK-2000SL, Metatek, Nonsan, Republic of Korea) at 50 °C for 30 min, 60 °C for 30 min, 70 °C for 60 min, and 75 °C for 120 min, followed by venting at 45 °C for 10 min and air cooling for 24 h. The finished jerky was packaged in 100 g portions and stored at 4 °C for use in experiments.

### 2.3. Meat Color and Sensory Evaluation

The color of the jerky was measured after exposing it to air for about 30 min using a colorimeter (CR-400, Konica Sensing Inc., Osaka, Japan) to measure CIE L* (lightness), a* (redness), and b* (yellowness) according to the Hunter scale. The instrument was standardized using a standard color plate (Y = 92.8, x = 0.3134, y = 0.3193) before measurement. Each experimental group was measured more than five times, and the average value was reported. Sensory evaluation was conducted by eight graduate students from the Department of Animal Resources at Daegu University, selected for their sensory discrimination ability. The panelists were trained three times and evaluated the samples using a 5-point scale method [24]. The quality evaluation of the jerky included assessments of color, flavor, off-flavor, juiciness, tenderness, and overall acceptability. A 5-point scale was used, where higher scores indicated better quality, except for off-flavor, where higher scores indicated stronger off-flavor. Juiciness was evaluated from very juicy (5) to not juicy (1), and tenderness from very tender (5) to very tough (1).

### 2.4. Volatile Basic Nitrogen (VBN), Lipid Peroxide, and Microbial Count during Storage

Thiobarbituric acid reactive substance (TBARS) values were assessed using the method outlined by Witte et al. [25]. In brief, 5 g of the sample was diluted with 45 mL distilled water and 50 μL BHA. The mixture was then blended for 1 min at maximum speed. Subsequently, 1 mL of the resulting blend was combined with 2 mL of TBA solution (consisting of 0.25 N HCl, 15% TCA, and 0.375% TBA reagent) and heated at 90 °C for 15 min. After centrifugation at 3100× *g* and 4 °C for 9 min, readings were obtained at 531 nm using an X-MA 4000 spectrophotometer (Human Co., Ltd., Gimpo, Republic of Korea). TBARS values were calculated as absorbance (O.D.) × 7.8 (mg of malondialdehyde (MDA)/kg of sample).

VBN contents were determined using Kang et al. [24]. Total aerobic bacteria (TAB) and coliform were assessed by plating the diluted homogenate onto total aerobic bacterial agar and McConkey sorbitol agar incubating at 37 °C for 24 h. Colony counts were expressed as log CFU/g.

### 2.5. Total Polyphenol and Flavonoid Content of BFE, JAE, and GEs

Total polyphenol content (TPC) was measured using a modified method by Rauha et al. [18]. Briefly, 100 μL of distilled water, 20 μL of sample extract, and 20 μL of Folin–Ciocalteu reagent (Sigma-Aldrich Co., St. Louis, MO, USA) were added to a 96-well plate. After 5 min, 10 μL of 20% sodium carbonate (Daejung Chemicals & Metals Co., Ltd., Siheung, Republic of Korea) was added, and the absorbance of the sample was measured at 715 nm using a spectrophotometer (Multiskan Go, Thermo Fisher Scientific Inc., Waltham, MA, USA). The amount was calculated from a standard calibration curve (*y* = 0.0001*x* + 0.0408, r^2^ = 0.9996) using catechin (Sigma-Aldrich Co., St. Louis, MO, USA). The results were expressed in mg of catechin equivalent (mg CAT eq/g extract). Total flavonoid content (TFC) was measured using a modified method by Kang et al. [24]. A mixture of 10 μL of the sample, 30 μL of methanol (Daejung Chemicals & Metals Co., Ltd., Siheung, Republic of Korea), 20 μL of 10% aluminum chloride (Sigma-Aldrich Co., St. Louis, MO, USA), 20 μL of 1% potassium acetate (Sigma-Aldrich Co., St. Louis, MO, USA), and 60 μL of 50% ethanol (Samchun Pure Chemical Co., Ltd., Seoul, Republic of Korea) was prepared. After standing for 30 min, the absorbance was measured at 415 nm using a spectrophotometer (Multiskan Go, Thermo Fisher Scientific Inc., Waltham, MA, USA). The sample was measured using a standard calibration curve (*y* = 0.0113*x* + 0.0444, r^2^ = 0.9974) with rutin (Sigma-Aldrich Co., St. Louis, MO, USA). The results were expressed in mg of rutin equivalent (mg RUT eq/g extract).

### 2.6. DPPH Radical Scavenging Activity and ABTS Radical Scavenging Activity

The antioxidant activity measurement using the 1,1-diphenyl-2-picrylhydrazyl (DPPH, Sigma-Aldrich Co., St. Louis, MO, USA) radical was conducted by modifying the method of Brand-Williams et al. [26]. To each sample (100 μg/mL, 100 μL), 100 μL of 0.1 mM DPPH ethanolic solution was added and mixed. The mixture was then allowed to stand for 1 h. After incubation, the absorbance of the sample was measured at 525 nm using a spectrophotometer (Multiskan Go, Thermo Fisher Scientific Inc., Waltham, MA, USA). The scavenging activity against DPPH was calculated using the following equation: Inhibition % = [(absorbance of control − absorbance of sample)/absorbance of control] × 100.

Antioxidant activity using the 2,2′-azino-bis (3-ethylbenzthiazoline-6-sulphonic acid) (ABTS, Sigma-Aldrich Co., St. Louis, MO, USA) radical was measured using a modified method of Kang et al. [24]. A 7 mM ABTS solution was mixed with 2.45 mM potassium persulfate (Sigma-Aldrich Co., St. Louis, MO, USA) and 95% ethanol to achieve an absorbance of 0.700 ± 0.020 nm. A mixture of 100 μL of sample and 100 μL of ABTS solution was prepared. After standing for 30 min, the absorbance was measured at 734 nm using a spectrophotometer (Multiskan Go, Thermo Fisher Scientific Inc., Waltham, MA, USA). The scavenging activity was calculated as follows: Inhibition% = [(absorbance of control−absorbance of sample)/absorbance of control] × 100.

### 2.7. Ferric-Reducing Antioxidant Power (FRAP)

The FRAP measurement using FeCl_3_ (Sigma-Aldrich Co., USA) was conducted using a modified method of Benzie and Strain [27]. FRAP reagent was prepared by mixing 0.3 M sodium acetate buffer (pH 3.6, Sigma-Aldrich Co., St. Louis, MO, USA), 10 mM 2,4,6-tripyridyl-S-triazine (Sigma-Aldrich Co., St. Louis, MO, USA) solution dissolved in 40 mM HCl (Samchun Pure Chemical Co., Ltd., Seoul, Republic of Korea), and 20 mM FeCl_3_ solution in a 10:1:1 (*v*/*v*/*v*) ratio. A mixture of 150 μL of FRAP reagent and 5 μL of sample was prepared. After standing at room temperature for 5 min, the absorbance was measured at 593 nm using a spectrophotometer (Multiskan Go, Thermo Fisher Scientific Inc., Waltham, MA, USA). The antioxidant power was expressed using a standard curve prepared with FeSO_4_·7H_2_O (0.1–1.0 mM) (Sigma-Aldrich Co., St. Louis, MO, USA).

### 2.8. Statistical Analysis

Data were presented as mean ± standard deviation (SD). Each experiment was repeated at least three times. Statistical analysis of the experimental results was performed using the General Linear Model (GLM) method of SAS 9.4 (SAS Institute Inc., Cary, NC, USA) [28]. The differences in results between treatments were confirmed by Duncan’s multiple-range test. Statistical significance was accepted at *p* < 0.05. Correlations among variables were performed with Pearson’s correlation test.

## 3. Results and Discussion

### 3.1. Total Polyphenol, Flavonoid Content, and Antioxidant Activity of Extracts

Table 2 shows the results of measuring the TPC and flavonoid content (TFC) of BFE, JAE, and GE. The TPC was adjusted to catechin and expressed as catechin equivalents (mg CAT eq/g extract). Phenolic compounds derived from natural sources are known for their antioxidant and free-radical scavenging activities [29,30]. The TPC of BFE was the highest at 6.85 mg CAT eq/g (*p* < 0.05), followed by GE and JAE at 2.86 and 1.75 mg CAT eq/g, respectively (*p* < 0.05). The TFC was adjusted to rutin and expressed as rutin equivalents (mg RUT eq/g extract). Similarly, the TFC was highest in BFE at 10.52 mg RUT eq/g (*p* < 0.05), followed by JAE and GE at 5.41 and 2.83 mg RUT eq/g, respectively (*p* < 0.05).

**Figure 1:**
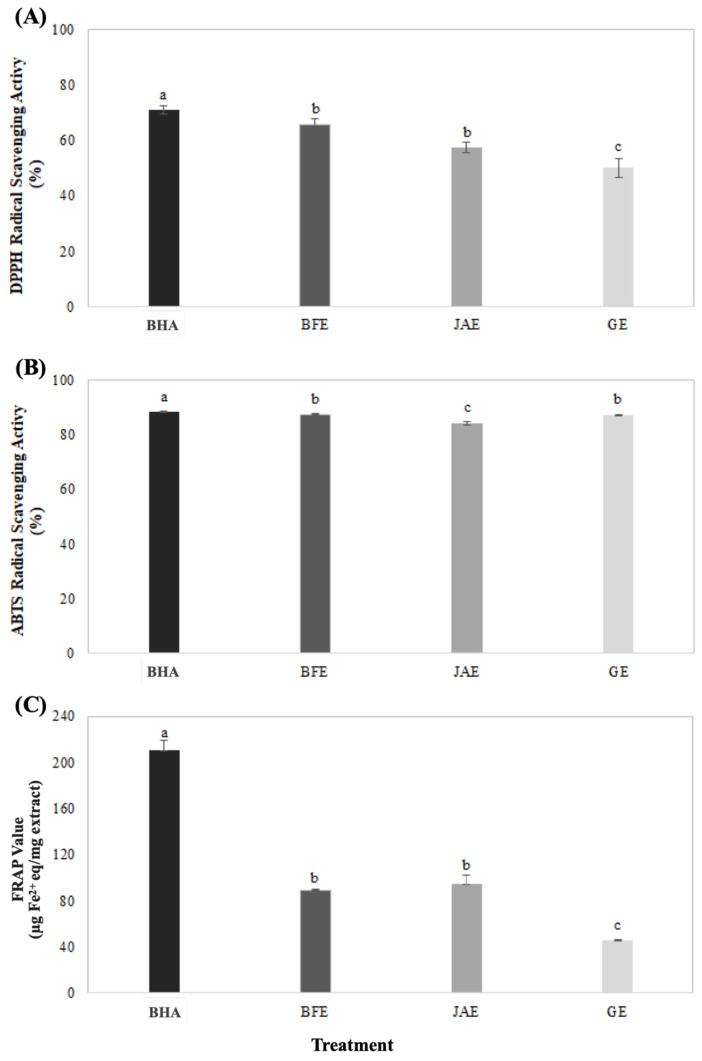
Radical scavenging activities of (**A**) DPPH and (**B**) ABTS and reducing power of (**C**) FRAP assay for BFE, JAE, and GE. BHA, butylated hydroxy anisole; BFE, balloon flower root; JAE, Japanese apricot; GE, grape extract. ^a–c^ Means with different superscripts in the values are significantly different at *p* < 0.05 by Duncan’s multiple range test.

These results suggest that these extracts could serve as natural antioxidants when added to jerky. The antioxidant activity values of the extracts are shown in Figure 1.

The DPPH radical scavenging activity, a method that measures the hydrogen-donating ability of antioxidants, showed that the positive control BHA exhibited the highest activity at 70.93% (*p* < 0.05) in Figure 1A. Among the extracts, BFE and JAE showed antioxidant activities of 62.96% and 57.45%, respectively, with no significant difference, while GE had the lowest activity at 50.02% (*p* < 0.05). For ABTS radical scavenging activity, BHA showed the highest activity at 88.36% (*p* < 0.05), with BFE and GE showing similar activities of 87.60% and 87.37%, respectively, and JAE showing the lowest activity at 84.42% (*p* < 0.05) in Figure 1B. Both DPPH and ABTS radical scavenging activities are related to phenolic compounds. These are known to contribute to antioxidant and antimicrobial activities, and various physiological functions [31,32]. The FRAP, which measures the electron-donating activity of antioxidants, showed that quercetin, the positive control, had the highest activity at 210.68 μg Fe^2+^ eq/mg (*p* < 0.05) in Figure 1C. Among the extracts, JAE and BFE showed similar activities at 94.26 and 88.69 μg Fe^2+^ eq/mg, respectively, with no significant difference, while GE had the lowest activity at 45.18 μg Fe^2+^ eq/mg (*p* < 0.05).

### 3.2. Yield of Jerky with Added Extracts

The production of other meat products, such as sausage, meat patty and ham, requires relatively lower temperatures and shorter processing times, while the production of jerky necessitates high drying temperatures and longer processing times. Therefore, the production process of jerky causes more physicochemical damage to the meat characteristics compared to the production processes of other meat products. Mick-lander et al. [31] found that at a heating temperature of approximately 40 °C, moisture migration and subsequent removal within the myofibrillar protein network was accelerated due to the denaturation of myosin heads, which reduces the myofibrillar protein network space. The yield of meat products is a critical factor affecting the productivity of the final product and is influenced by protein denaturation and water retention during heating [33]. The yield for jerky prepared with curing solutions containing PS, VE, BFE, JAE, and GEs ranged from 39.51% to 43.60% with no significant differences observed (Table 3). After drying at 75 °C for 120 min, the difference in yield of the beef jerky with different additives was not statistically significant (*p* > 0.05). These results indicate that the addition of BFE, JAE, and GE did not affect the yield of the beef jerky and that they did not negatively affect moisture loss or protein degradation during the manufacturing process.

### 3.3. Meat Color of Jerky with Added Extracts

The color of meat products is a critical meat quality attribute that significantly influences consumer perception and purchase decisions [33,34,35]. Color is also a critical quality attribute in meat products that reflects changes in appearance due to various factors [34]. Therefore, understanding the factors that contribute to variations in meat color is of paramount importance for the meat industry to effectively meet market demands and ensure consumer satisfaction. Several studies have documented the beneficial effects of novel ingredients such as antioxidants, stabilizers, and probiotics in improving the physicochemical properties, nutritional composition, and safety profile of beef jerky [36,37]. Table 4 shows the color changes in beef jerky during storage with added extracts. On day 1 of storage, the JAE and GE treatment groups showed significantly lower L* values compared to the PS group (*p* < 0.05), but the BFE treatment group did not show significant differences compared to PS and VE treatment groups (*p* > 0.05). These results suggest that BFE treatment did not reduce the lightness of beef jerky. During the drying process, the color changes were related to myoglobin oxidation [38] due to the inevitable oxidation of heme. Therefore, the heme in myoglobin gradually decreases the content of oxymyoglobin (bright red) content and increases the content of metmyoglobin (brown red) content [39,40]. On day 1 of storage, the a* (redness) values were lower in the JAE treatment group than in the PS and VE treatment groups (*p* < 0.05). However, BFE and GE treatment groups showed no significant color differences compared to PS and VE treatment groups on day 1 of storage, indicating that BFE and GE did not reduce the redness of beef jerky. In b* (yellowness) values, the BFE, JAE, and GE treatment groups showed significantly lower values compared to the PS and VE treatment groups (*p* < 0.05) at day 1 of storage, indicating their superior performance in yellowness. At day 30 of storage, no significant differences in the L* values were observed among the groups, and the a* values of BEF and PS treatment groups were higher than the other groups (*p* < 0.05). The b* values of the JAE, GE, and VE treatment groups were significantly lower than those of the PS and BFE treatment groups at day 30 of storage (*p* < 0.05). Similarly, *P. mume* treatment in chicken did not change color values [41]. The grape seed extract also did not alter color [42].

### 3.4. The TBARS, VBN and Microbial Values of Jerky with Added Extracts

VBN and lipid peroxide content are shown in Table 5. VBN content increased over the storage period in jerky with PS, JAE, and GEs (*p* < 0.05), while no significant difference was observed in jerky with VE and BFE (*p* > 0.05). Lipid peroxide levels were lowest in jerky with BFE at day 1 (*p* < 0.05) and with PS, VE, and GEs at day 30 compared to another jerky (*p* < 0.05). The results of this study indicated that on day 30 of storage, the addition of GE extract, similar to the treatment groups of PS and VE, resulted in significantly lower TBARS values compared to the BFE and JAE groups (*p* < 0.05). These findings suggest that the inclusion of 0.05% grape extract in beef jerky effectively suppressed lipid oxidation, yielding values similar to those obtained with the commercially used 0.10% PS and 0.05% VE treatments, within the range of 0.368–0.371 mg MDA/g. These values are considered to be significantly favorable and superior, indicating positive results in terms of oxidation inhibition of meat products [43]. Similar trends were noted, such as *P. grandiflorum* (0.1%) was effective in reducing lipid oxidation in sheep meat [44]. *P. mume* jerky showed lower TBARS values than control and this could be because it had antioxidative and free-radical scavenging activity as it contains flavonoids such as naringenin and rutin [6]. Similar data found that beef sausages containing 0.05% grape seed extract reduced lipid oxidation due to its high content of polyphenolic compounds and proanthocyanidins [45]. In pork liver pate, the addition of grape seed extract (1000 mg/kg) improved the oxidative stability more than the treatment added with BHT (200 mg/kg) during storage [46].

The microbial analysis results are shown in Table 6. Total plate counts on day 1 were 1.23 log CFU/g and 1.19 log CFU/g for jerky with PS and GE, respectively, but were not detected on day 30. No coliform bacteria were detected on both day 1 and day 30. The results of this study, which showed that total bacteria count and *E. coli* were undetectable on day 30 of storage, are considered highly significant for the hygienic quality and extended shelf life of jerky. Luo et al. [44] also reported that *P. grandiflorum* (0.1%) had an antimicrobial effect in sheep meat. GE has shown excellent antibacterial activity against various pathogenic bacteria in other studies [45].

### 3.5. The Sensory Quality of Jerky with Added Extracts

The sensory evaluation results are shown in Table 7. In sensory analysis, at days 1 and 30 of storage, there was no significant difference between treatments in terms of flavor, off-flavor, and beef jerky juiciness. For color preference, there was no significant difference between treatments on day 1 of storage. However, at day 30 of storage, the BFE treatment group exhibited significantly higher color preferences compared to other groups (*p* < 0.05), while the GE treatment group showed significantly lower color preferences (*p* < 0.05). The correlation between color preference and the instrumental measurements of L*, a*, and b* values were r = 0.39 (*p* > 0.05), r = 0.68 (*p* < 0.01), and r = 0.81 (*p* < 0.01), respectively. These results indicate that the BFE group, with relatively high a* and b* values, had a higher color preference. In contrast, the GE group, with relatively low a* and b* values, showed lower color preference. In terms of tenderness of beef jerky, the GE extract-treated group showed significantly higher tenderness than other jerky on day 1 of storage (*p* < 0.05); however, no significant difference was observed in tenderness between samples at day 30 (*p* > 0.05). For overall acceptability in sensory analysis, the BFE treatment group consistently received higher scores than the other treatments throughout the storage period (*p* < 0.05). Luo et al. [44] also noted that sheep meat with BFE increased meat redness compared to the control samples.

### 3.6. Correlation Analysis

This study correlates the total phenolic and flavonoid contents and antioxidant activities of the selected extracts. The relationship was calculated using Pearson’s correlation. The total phenolics negatively correlated with total flavonoids (r = −0.54, *p* < 0.05), meaning that the higher the total phenolic content, the lower the TFC. It is reported that the extraction yields of phenolic compounds and flavonoids from plant materials can significantly differ due to the polarity of the solvents used [47,48]. These results have been reported because TPC shows higher extraction efficiency with high-polarity solvents like water or methanol, while TFC is better extracted with moderately polar or non-polar solvents such as ethanol or acetone [49]. In general, total phenolics and flavonoids play an important role in antioxidant activity acting as free-radical chain terminators, free-radical scavengers, and electron donors [50]. Table 8 shows a positive correlation between TPC vs. DPPH and TFC vs. DPPH with r = 0.93 and r = 0.65, respectively (*p* < 0.01). Furthermore, a moderate positive correlation was observed between ABTS vs. TPC and ABTS vs. TFC with r = 0.68 (*p* < 0.01) and r = 0.58 (*p* < 0.05), respectively, but ABTS had a weak correlation with DPPH (r = 0.23, *p* < 0.05). TPC and FRAP had a weak correlation (r = 0.25, *p* < 0.05), which shows a weak relationship between phenolics and Fe^3+^ reduction activity. In contrast, a moderate positive correlation was identified for TFC vs. FRAP (r = 0.69, *p* < 0.01). The higher the flavonoid content, the higher the reduction activity. This indicated that the flavonoid compounds contained in the extracts were directly proportional to the reduction power. To perform a correlation analysis of TBARSs with other parameters (TPC, TFC, DPPH, ABTS, and FRAP), TBARS values from the 30-day storage data were used. The TBARS was positive correlated with TPC and DPPH (r = 0.64, *p* < 0.01 and r = 0.75, *p* < 0.01). Meanwhile the weak correlations between TBARSs with TFC, ABTS and FRAP were valued at r = 0.27, r = 0.40 and r = 0.29, respectively (*p* < 0.05). These results suggest that the lipid oxidation of beef jerky is highly correlated with the TPC and DPPH activities of the extracts. This result was similar to the finding that phenolic compounds with low molecular weight in the extract exhibited higher DPPH activity compared to flavonoids [51].

## 4. Conclusions

The extracts were successfully prepared and utilized in the production of beef jerky. The extracts were found to have remarkable antioxidant activity, which may contribute to the preservation and flavor of the jerky. BFE exhibited the highest polyphenol and flavonoid content. The yield of the jerky produced using these extracts was found to be comparable to that of the control group, which used conventional preservatives. This suggests that the utilization of these natural extracts does not negatively impact the productivity of jerky production. The color of the jerky was not significantly affected by the extracts, except for an increased redness observed in jerky treated with BFE. The increased redness may potentially enhance the visual appeal of the product to consumers. The lowest levels of lipid peroxide were observed in the jerky samples treated with BFE at day 1 and with PS, VE, and GEs at day 30. This suggests that these extracts can effectively reduce lipid oxidation in the jerky, thereby potentially extending its shelf life. The microbial analysis demonstrated that the total plate counts were low on day 1 and not detected on day 30, indicating that the use of these extracts does not compromise the microbial safety of the jerky. Additionally, the incorporation of BFE, JAE, and GEs into the jerky enhanced its shelf life and sensory properties. Notably, BFE and GEs demonstrated potential as viable alternatives to synthetic preservatives. Further investigation is necessary to elucidate the biological impacts of natural extracts such as BFE, JAE, and GE in the meat industry.

## Figures and Tables

**Table 1:** The formula for added-to-water extracts of balloon flower root (*Platycodon grandiflorum* (JACQ.) A. DC.), Japanese apricot (*Prunus mume Sieb.* et Zucc) and grape (*Vitis vinifera*) on beef jerky.

Ingredients	Treatments ^1^				
	PS	VE	BFE	JAE	GE
Beef	84.000	84.000	84.000	84.000	84.000
Soy sauce	2.000	2.000	2.000	2.000	2.000
Sodium nitrite	0.008	0.008	0.008	0.008	0.008
Ascorbic acid	0.250	0.250	0.250	0.250	0.250
Salt	0.500	0.500	0.500	0.500	0.500
Sodium polyphosphate	0.170	0.170	0.170	0.170	0.170
Sugar	4.570	4.570	4.565	4.570	4.565
Beef powder	0.560	0.560	0.560	0.560	0.560
Bulgogi spice	2.000	2.000	2.000	2.000	2.000
Black pepper	0.450	0.450	0.450	0.450	0.450
Sodium L-glutamate	0.460	0.460	0.460	0.460	0.460
Onion powder	0.300	0.300	0.300	0.300	0.300
Garlic powder	0.300	0.300	0.300	0.300	0.300
Sausage spice	0.230	0.230	0.230	0.230	0.230
D-sorbitol	1.500	1.500	1.500	1.500	1.500
Water	2.602	2.652	2.657	2.652	2.657
Total	99.900	99.950	99.950	99.950	99.950
PS	0.100	NA	NA	NA	NA
VE	NA	0.050	NA	NA	NA
BFE	NA	NA	0.050	NA	NA
JAE	NA	NA	NA	0.050	NA
GE	NA	NA	NA	NA	0.050

^1^ PS, potassium sorbate (0.10%); VE, vitamin E (0.05%); BFE, balloon flower root extract (0.05%); JAE, Japanese apricot extract (0.05%); GE, grape extract (0.05%); and NA, not applicable.

**Table 2:** Total polyphenol and total flavonoid contents of BFE, JAE, and GE.

Extracts ^1^	TPC ^2^ (mg CAT eq/g Extract) ^3^	TFC ^2^ (mg RUT eq/g Extract) ^3^
BFE	6.85 ± 0.19 ^a^	10.52 ± 0.22 ^a^
JAE	1.75 ± 0.12 ^c^	5.41 ± 0.35 ^b^
GE	2.86 ± 0.25 ^b^	2.83 ± 1.07 ^c^
SEM ^4^	0.20	0.66

^1^ BFE, balloon flower root; JAE, Japanese apricot; GE, grape extract. ^2^ TPC, total polyphenol content; TFC, total flavonoid content. ^3^ CAT eq: cathechin equivalents; RUT eq: rutine equivalents. ^4^ SEM: standard error of means. ^a–c^ Values with different superscripts in the same column are significantly different at *p* < 0.05 by Duncan’s multiple range test.

**Table 3:** Yield of beef jerky added with BFE, JAE, and GE.

Treatments ^1^	Yield (%)
PS	42.11 ± 1.61
VE	40.67 ± 0.97
BFE	41.04 ± 2.08
JAE	39.51 ± 1.14
GE	43.60 ± 0.62
SEM ^2^	1.38

^1^ PS, potassium sorbate (0.10%); VE, vitamin E (0.05%); BFE, balloon flower root (0.05%); JAE, Japanese apricot (0.05%); GE, grape extract (0.05%). ^2^ SEM: standard error of means.

**Table 4:** The effect of BFE, JAE, and GE on the surface color values of beef jerky during storage.

Color ^1^	Storage (Days)	Treatments ^2^					SEM ^3^
		PS	VE	BFE	JAE	GE	
L*	1	40.81 ± 1.45 ^aA^	39.07 ± 1.95 ^bcA^	39.32 ± 1.05 ^abA^	35.89 ± 0.62 ^dA^	37.51 ± 0.64 ^cdA^	1.25
	30	26.81 ± 3.08 ^B^	26.18 ± 1.15 ^B^	26.97 ± 1.20 ^B^	24.42 ± 1.96 ^B^	24.94 ± 1.63 ^B^	1.93
a*	1	10.04 ± 1.31 ^aB^	10.37 ± 1.39 ^aB^	8.26 ± 1.03 ^bcB^	7.19 ± 0.86 ^cB^	9.43 ± 0.62 ^abB^	1.08
	30	18.84 ± 2.37 ^aA^	16.15 ± 2.38 ^bA^	19.63 ± 1.71 ^aA^	16.22 ± 1.46 ^bA^	13.21 ± 1.14 ^cA^	1.88
b*	1	5.73 ± 0.98 ^aB^	5.37 ± 1.66 ^aB^	3.60 ± 0.55 ^bB^	3.20 ± 0.62 ^bB^	3.61 ± 0.15 ^bB^	0.94
	30	12.88 ± 2.06 ^bA^	7.91 ± 0.40 ^cA^	14.80 ± 0.32 ^aA^	8.01 ± 1.43 ^cA^	8.53 ± 1.14 ^cA^	1.50

^1^ L*, brightness/darkness; a*, (+) redness/(–) greenness; and b*, (+) yellowness/(–) blueness. ^2^ PS, potassium sorbate (0.10%); VE, vitamin E (0.05%); BFE, balloon flower root (0.05%); JAE, Japanese apricot (0.05%); GE, grape extract (0.05%). ^3^ SEM: standard error of means. ^a–d^ Values (mean ± SD) with different superscripts in the same column are significantly different at *p* < 0.05 by Duncan’s multiple range test. ^A,B^ Values (mean ± SD) within a row with different superscripts are significantly different at *p* < 0.05 by Duncan’s multiple range test.

**Table 5:** The effect of BFE, JAE, and GE on TBARS (mg MDA/kg) and VBN (mg%) values of beef jerky during storage.

Trait	Storage (Days)	Treatments ^1^					SEM ^2^
		PS	VE	BFE	JAE	GE	
TBARS	1	0.272 ± 0.001 ^aB^	0.263 ± 0.002 ^bB^	0.255 ± 0.001 ^cB^	0.263 ± 0.002 ^bB^	0.261 ± 0.002 ^bB^	0.00
	30	0.371 ± 0.004 ^cA^	0.368 ± 0.003 ^cA^	0.381 ± 0.001 ^bA^	0.427 ± 0.005 ^aA^	0.371 ± 0.005 ^cA^	0.01
VBN	1	9.34 ± 0.81 ^bB^	12.89 ± 0.49 ^a^	13.63 ± 0.32 ^a^	10.27 ± 0.81 ^bB^	12.14 ± 1.62 ^aB^	0.92
	30	11.86 ± 0.61 ^dA^	12.51 ± 0.16 ^c^	14.47 ± 0.43 ^b^	15.13 ± 0.28 ^aA^	15.22 ± 0.32 ^aA^	0.29

TBARS, thiobarbituric acid reactive substance; MDA, malondialdehyde; VBN, volatile basic nitrogen. ^1^ PS, potassium sorbate (0.10%); VE, vitamin E (0.05%); BFE, balloon flower root (0.05%); JAE, Japanese apricot (0.05%); GE, grape extract (0.05%). ^2^ SEM: standard error of means. ^a–c^ Values (mean ± SD) with different superscripts in the same column are significantly different at *p* < 0.05 by Duncan’s multiple range test. ^A,B^ Values (mean ± SD) within a row with different superscripts are significantly different at *p* < 0.05 by Duncan’s multiple range test.

**Table 6:** The effect of BFE, JAE, and GE on the total microbial counts (TPC, log CFU/g) and *E. coli* counts (log CFU/g) of beef jerky during storage.

Trait	Storage (Days)	Treatments ^1^					SEM ^2^
		PS	VE	BFE	JAE	GE	
TPC	1	1.23 ± 0.09 ^A^	ND	ND	ND	1.19 ± 0.11 ^A^	0.02
	30	ND ^B^	ND	ND	ND	ND ^B^	NA
*E. coli*	1	ND	ND	ND	ND	ND	NA
	30	ND	ND	ND	ND	ND	NA

^1^ PS, potassium sorbate (0.10%); VE, vitamin E (0.05%); BFE, balloon flower root (0.05%); JAE, Japanese apricot (0.05%); GE, grape extract (0.05%). ^2^ SEM: standard error of means. Values (mean ± SD) with different superscripts in the same column are significantly different at *p* < 0.05 by Duncan’s multiple range test. ^A,B^ Values (mean ± SD) within a row with different superscripts are significantly different at *p* < 0.05 by Duncan’s multiple range test. ND, not detected; NA, not applicable.

**Table 7:** The effect of BFE, JAE, and GE on the sensory quality of beef jerky during storage.

Sensory Quality	Storage (Days)	Treatments ^1^					SEM ^2^
		PS	VE	BFE	JAE	GE	
Color	1	3.50 ± 0.50	3.60 ± 0.53 ^A^	3.43 ± 0.40 ^B^	3.27 ± 0.25	3.07 ± 0.12 ^A^	0.39
	30	3.77 ± 0.25 ^b^	3.20 ± 0.17 ^cdB^	4.20 ± 0.17 ^aA^	3.57 ± 0.25 ^bc^	2.97 ± 0.15 ^dB^	0.20
Flavor	1	3.87 ± 0.32	3.77 ± 0.25	4.13 ± 0.21	4.00 ± 0.10	3.93 ± 0.15 ^B^	0.22
	30	3.90 ± 0.10 ^b^	3.45 ± 0.10 ^c^	4.27 ± 0.25 ^a^	4.03 ± 0.06 ^ab^	4.10 ± 0.17 ^abA^	0.20
Off-flavor	1	1.03 ± 0.06	1.03 ± 0.06	1.00 ± 0.00	1.00 ± 0.00	1.00 ± 0.00	0.04
	30	1.07 ± 0.06	1.00 ± 0.00	1.00 ± 0.00	1.03 ± 0.06	1.03 ± 0.06	0.05
Juiciness	1	3.70 ± 0.17	3.67 ± 0.29	3.60 ± 0.17	3.37 ± 0.12	3.57 ± 0.21	0.20
	30	3.60 ± 0.17	3.60 ± 0.10	3.70 ± 0.36	3.83 ± 0.29	3.60 ± 0.17	0.24
Tenderness	1	3.20 ± 0.17 ^bc^	2.97 ± 0.15 ^c^	3.20 ± 0.26 ^bc^	3.47 ± 0.15 ^b^	3.83 ± 0.15 ^a^	0.18
	30	3.13 ± 0.15	3.43 ± 0.40	3.57 ± 0.40	3.10 ± 0.10	3.30 ± 0.20	0.28
Overall acceptability	1	3.30 ± 0.20 ^c^	3.40 ± 0.20 ^bc^	4.33 ± 0.15 ^a^	3.70 ± 0.26 ^bc^	3.73 ± 0.25 ^b^	0.22
	30	3.23 ± 0.20 ^b^	3.43 ± 0.38 ^b^	4.46 ± 0.21 ^a^	3.53 ± 0.45 ^b^	3.76 ± 0.21 ^b^	0.31

^1^ PS, potassium sorbate (0.10%); VE, vitamin E (0.05%); BFE, balloon flower root (0.05%); JAE, Japanese apricot (0.05%); GE, grape extract (0.05%). ^2^ SEM: standard error of means. ^a–d^ Values (mean ± SD) with different superscripts in the same column are significantly different at *p* < 0.05 by Duncan’s multiple range test. ^A,B^ Values (mean ± SD) within a row with different superscripts are significantly different at *p* < 0.05 by Duncan’s multiple range test.

**Table 8:** Pearson’s correlation coefficient of TPC and TFC of extracts with their DPPH and ABST radical scavenging activities and FRAP values.

	TFC	DPPH	ABTS	FRAP	TBARS
TPC	–0.54 *	0.93 **	0.68 **	0.25 *	0.67 **
TFC		0.65 **	0.58 **	0.69 **	0.17 *
DPPH			0.23 *	0.39 *	0.75 **
ABTS				0.29	0.40 *
FRAP					0.29 *

* Correlation coefficient is significant at *p* < 0.05; ** Correlation coefficient is significant at *p* < 0.01.

## Data Availability

The original contributions presented in the study are included in the article; further inquiries can be directed to the corresponding author.
